# Expanded newborn screening for inborn errors of metabolism and genetic variants in Xinjiang, China

**DOI:** 10.3389/fgene.2025.1617418

**Published:** 2025-07-14

**Authors:** Huiwen Zhang, Haoyue Tian, Wencheng Dai, Luhan Zhang, Gang Guo, Guifeng Ding

**Affiliations:** ^1^ College of Public Health, Xinjiang Medical University, Urumqi, China; ^2^ Prenatal Diagnosis Center, Urumqi Maternal and Child Health Hospital, Urumqi, China; ^3^ Xinjiang Clinical Research Center for Perinatal Diseases, Urumqi Maternal and Child Health Hospital, Urumqi, China

**Keywords:** inborn errors of metabolism, newborn screening, tandem mass spectrometry, next-generation sequencing, genetic variants

## Abstract

Inborn errors of metabolism (IEMs), with diverse clinical phenotypes, are featured primarily by complex etiology, lack of specificity in clinical manifestations, major damage to the nervous and digestive system, and even death, bringing great pain and economic burden to children and families. Expanded newborn screening for IEMs by tandem mass spectrometry (MS/MS) is effective to realize early diagnosis and presymptomatic treatment, which may be useful for preventing severe permanent sequelae and death. This study was scheduled to determine the disease spectrum, prevalence and gene variants of IEMs in Xinjiang, China. A sum of 107,741 newborns were screened by MS/MS from January 2019 to December 2024. After initial screening, 3947 newborns, who had positive results, needed to be recalled. The number of successful recalls was 3817 and the recall rate was 96.71%. Suspected positive patients were further diagnosed through next-generation sequencing (NGS) and validated by sanger sequencing. Seventy-three patients were diagnosed with IEMs in Xinjiang, resulting in an overall incidence of 1/1,476. The incidence of amino acid, organic acid, and fatty acid metabolic disorders were 1/1995, 1/8978 and 1/15392, respectively. Hyperphenylalaninemia, Hypermethioninemia and Methylmalonic acidemia ranked the top 3 of all detected IEMs. One hundred twenty-seven mutations in eleven IEMs-associated genes were identified in 69 confirmed cases. Several hotspot mutations in the *PAH* gene were identified. This study observed some unreported mutation sites in *MAT1A* gene, which enriched the gene database. Therefore, our data clearly elucidated the disease spectrum and genetic variants in Xinjiang, contributing to the treatment and prenatal genetic counseling of these disorders in this region.

## Introduction

Inborn errors of metabolism (IEMs) is defined as a group of disorders in which the genes coding for enzymes, carrier proteins, membrane proteins or receptors involved in metabolism undergo mutations that cause structural or functional changes of relevant proteins, resulting in metabolic disorders as well as corresponding physiopathological changes and clinical manifestations. The majority of IEMs are autosomal recessive disorders, including amino acidemias, organic acidemias, and fatty acid oxidation disorders (Ferreira and van Karnebeek, 2019). IEMs include over 1,000 types of diseases, 500–600 types of which are common in the world (Hao and Xiao, 2019). IEMs cause significant suffering and impose a heavy economic burden on affected children and their families. Tandem mass spectrometry (MS/MS) is an efficient biometabolite detection with high specificity and sensitivity, capable of detecting dozens of metabolites in a sample within 2–3 min, facilitating the detection and analysis of a series of IEMs (Zhao et al., 2022). Next-generation sequencing (NGS) has been accepted to be a fast-developing technology for IEMs diagnosis, providing an important means for confirming the diagnosis of suspected positive children screened by MS/MS, leading to an effective reduction in child disability and mortality (Tang et al., 2024).

In view of the above, this study investigated 107,741 newborns who underwent MS/MS screening at Urumqi Maternal and Child Healthcare Hospital from January 2019 to December 2024. This study primarily aimed to determine the prevalence and spectrum of IEMs in newborns in Xinjiang using MS/MS. Additionally, it sought to characterize gene variants via NGS, evaluate mutation distribution, and provide data to support the local prevention and control strategy for IEMs.

## Materials and methods

### Subjects

From January 2019 to December 2024, the dried blood spot samples prepared from the heel blood of 107,741 newborns from four Xinjiang regions (Urumqi, Changji, Turpan and Tacheng regions) were sent to Urumqi Maternal and Child Healthcare Hospital for MS/MS screening. Guardians of the newborns signed the informed consent for newborn screening for IEMs. This study has been approved by the Medical Ethics Committee of Urumqi Maternal and Child Health Hospital (XJFYLL2025006).

### Inclusion and exclusion criteria

#### Inclusion criteria

Newborns who are 72 h old and have been exclusively breastfed; Families of newborns have signed a written informed consent form.

#### Exclusion criteria

Newborns whose families explicitly refuse to participate in the screening after receiving education; Newborns who fail to exhibit spontaneous breathing or heartbeats immediately after resuscitation, or who do not show spontaneous breathing after birth; Other newborns for whom blood samples were not collected within the specified time frame.

### Instruments and reagents

Waters Xevo TQD tandem mass spectrometer (United States), ALLSHENG microplate constant temperature incubation oscillator, Eppendorf pipette, Henya ultrasonic cleaning machine. The non-derivatization tandem mass spectrometry screening kit (NeoBase™ Non-derivatizad MS/MS Kit) and supporting reagents, including extraction solutions and mobile phases, were produced by Perkin Elmer (United States). The purity of the organic solvents (such as methanol) used in the experiment was all high-performance liquid chromatography (HPLC) grade. The argon gas required for the MS crash cell was purchased from a local gas company, with a purity >99.999%.

### MS/MS

All related procedures were completed followed the Technical Specifications for Neonatal Disease Screening (2010 Edition) (Ministry of Health of the People’s Republic of China, 2010). Newborns were fully breastfed at 72 h after birth for the harvesting of their heel blood samples with a special filter paper sheet. At least three blood spots, each with a diameter of at least 8 mm, were collected. Following air-drying, the blood specimen was placed into a sealed, airtight bag and stored in a refrigerator maintained at 2°C–8°C. Then, the dried blood sheet was delivered to the Neonatal Disease Detection Laboratory within 1 week. Dried blood spots were processed under the instruction by the product manual of NeoBase™ non-derivatizad MS/MS Kit. Dried blood spot samples and quality control blood spots (with high and low concentrations) were punched with a 3.2 mm diameter punching pliers. One blood spot was placed into each well of a V-shaped cut-off bottom 96-well plate. After that, 1 mL of extraction solution was added into the vials of dry powder of amino acid and carnitine internal standards respectively. When sealed with sealing film, the vials placed into the sonicator for 30 min, and then fixed in the vortex oscillator to oscillate until completely dissolved. After these steps, the vials were stored in the refrigerator at 2°C–8°C. Furthermore, the re-solubilized amino acid and carnitine standards were removed from the refrigerator and returned to room temperature in dark, after which, they were shaken with a vortex oscillator for 2 min, followed by centrifugation at low speed for 1 min, and final dilution with the extraction solution at 1:110. Next, 100 μL of the extract solution was added into each well with a multichannel pipette, then the plate was sealed with an adhesive sealing film for placement into a thermostatic shaking incubator at 45°C and 700 r/min for 45 min. Following the removal of the adhesive sealing film, 75 μL of supernatant was aspirated from each well with a multichannel pipette, transferred to a 96-well V-bottom heat-resistant plate, with the whole plate covered by aluminum foil, and tested on the machine.

### Genetic analysis

Genomic DNA was extracted from 2 mL venous blood samples of children and their parents using the CWE9600 Blood DNA Kit (CWBIOTECH). Qubit was used for DNA sample quality control. Then, samples were placed in the refrigerator at −20°C.

DNA library preparation was performed, including end repair, connector connection, and polymerase chain reaction amplification. After library construction, samples were quantified and diluted according to the kit protocol, after which sequencing was conducted using the sequencing platform. Sequencing bioinformatics analysis included using the Burrow-Wheeler Aligner (BWA) sequence alignment method and the quality control sequence contrast human genome reference sequence. Finally, the pathogenicity of the mutations was analyzed according to the guidelines of the American College of Medical Genetics and Genomics (Richards et al., 2015) in the light of the conditions of the affected children. Furthermore, the positive mutation sites in the high-throughput sequencing results were verified based on the conventional Sanger sequencing, which was also applied to the genomic DNA of the parents of the children to detect the corresponding positive sites. In this study, the genetic tests were completed by Shanghai Xinhua Hospital, Shaanxi Baimei Medical Laboratory, and Hangzhou Bosheng Medical Laboratory.

### Quality control

The Neonatal Disease Detection Laboratory has provided training for all blood collection staff within its jurisdiction. The trained blood collection staff are responsible for the collection of neonatal heel blood, information entry and blood film delivery. The collected filter paper dried blood smear specimens were transported under cold chain throughout the process, thus ensuring the quality of screening. Regular maintenance and upkeep of instruments and equipment. The inter-laboratory quality assessment of MS/MS screening of neonatal genetic metabolic diseases was organized by the Clinical Laboratory Center of the National Health Commission every year and all indicators were 100% qualified.

### Statistical analysis

All data obtained in our study were processed by SPSS 26.0 and descriptive statistics. The difference of categorical data was compared using Chi-square test. The difference of measurement data was compared by analysis of variance. Values were considered statistically significant when *p* < 0.05. Counting data were expressed as number of cases and rate or composition ratio (%).

## Results

### Incidence and characteristics of IEMs

From January 2019 to December 2024, a total of 107,741 newborn babies were screened by MS/MS, including 55,516 males and 52,225 females; the percentage of preterm infants was 6.81% (7,332/107,741) and that of low-birth-weight infants was 4.14% (4,462/107,741). The characteristics of newborn screening were detailed in Table 1. After initial screening, 3947 newborns, who had positive results, needed to be recalled. The number of successful recalls was 3817 and the recall rate was 96.71%. Finally, 73 infants were diagnosed with one of IEMs and treated, and 69 of them were referred to genetic analysis. A total of twelve different IEMs, three were amino acidemia (54 cases; 73.97%), six were organic acidemia (12 cases; 16.44%) and three were fatty acid oxidation disorders (7 cases; 9.59%). The incidence of amino acidemia was 1/1,995, of which there were forty-seven cases of Phenylalanine hydroxylase deficiency (PAHD), six cases of Hypermethioninemia (MIM), and one case of Maple syrup urine disease (MSUD). PAHD was the most common disease for amino acidemia (1/2,292). The mean primary screening concentrations of Phe was 161.40 μmol/L and Phe/Tyr was 1.75 μmol/L.

**TABLE 1:** Baseline characteristics of newborn babies in the expanded newborn screening program.

General data grouping	Newborns without IEM N = 107668	Newborns with a confirmed IEM N = 73	P
Maternal age at delivery (years)	30.01 ± 4.435	30.29 ± 4.492	0.593
Newborn sex			
Male	55481	35	0.540
Female	52187	38	
Gestational age (weeks)			
<32	454	1	0.621
32–36^+6^	6874	3	
≥37	100340	69	
Birth weight(g)			
<2500	4459	3	0.966
2500–3999	92116	63	
≥4000	11093	7	
Mode of delivery			
Spontaneous labor	51021	36	0.742
Cesarean section	56647	37	

Twelve cases of organic acidemia (1/8,978) were reported, of which methylmalonic acidemia (MMA) was the highest (1/21,548); besides, there were three cases of 3-methylcrotonyl CoA carboxylase deficiency (3-MCCD), one case of propionic acidemia (PA), one case of isovaleric acidemia (IVA), one case of glutaric acidemia type I deficiency (GA-I) and one case of 2-methylbutyryl-CoA dehydrogenase deficiency (2-MBAD). Fives cases of MMA included three cases of pure MMA and two cases of MMA combined with homocysteinemia. The average primary screening concentration of C3, C3/C0, and C3/C2 in all patients with MMA were 16.74, 0.61 and 0.60 μmol/L, respectively. Seven cases of fatty acid metabolism disorder (1/15,392) were found,of which short chain acyl-CoA dehydrogenase deficiency (SCADD) was the highest (1/35,914); besides, there were two cases of Medium chain acyl-CoA dehydrogenase deficiency (MCADD) and two cases of Primary carnitine deficiency (PCD). Children with PCD showed an decrease of C0 detected by MS/MS. After clinical administration of levocarnitine supplementation, C0 returned to normal. The children has good growth now. All the above data were shown in Tables 2, 3.

**TABLE 2:** The spectrum and incidence of conditions from 107,741 newborns screened by expanded newborn screening program.

Conditions	Patients	Estimated incidence	95%CI
**Amino acid disorders (AAD)**	**54**	**1/1,995**	1/2,505–1/1,607
Phenylalanine hydroxylase deficiency (PAHD)	47	1/2,292	1/3,207–1/1,663
Hypermethioninemia (MIM)	6	1/17,957	1/48,980–1/8,263
Maple syrup urine disease (MSUD)	1	1/107,741	1/4,257,000–1/19,343
**Organic acid disorders (OAD)**	**12**	**1/8,978**	1/14,784–1/5,709
Methylmalonic acidemia (MMA)	5	1/21,548	1/43,096–1/8,619
3-methylcrotonyl CoA carboxylase deficiency (3-MCCD)	3	1/35,914	1/106,667–1/10,866
Propionic acidemia (PA)	1	1/107,741	1/4,257,000–1/19,343
Isovaleric acidemia (IVA)	1	1/107,741	1/4,257,000–1/19,343
Glutaric acidemia type I deficiency (GA-I)	1	1/107,741	1/4,257,000–1/19,343
2-methylbutyryl-CoA dehydrogenase deficiency (2-MBAD)	1	1/107,741	1/4,257,000–1/19,343
**Fatty acid oxidation disorders (FAOD)**	**7**	**1/15,392**	1/26,953–1/8,543
Short chain acyl-CoA dehydrogenase deficiency (SCADD)	3	1/35,914	1/106,667–1/10,866
Medium chain acyl-CoA dehydrogenase deficiency (MCADD)	2	1/53,871	1/215,482–1/15,392
Primary carnitine deficiency (PCD)	2	1/53,871	1/215,482–1/15,392

Note: IEMs comprise three main categories: amino acid disorders, organic acid disorders and fatty acid oxidation disorders.

**TABLE 3:** Results of tandem mass spectrometry in 73 children with genetic metabolic diseases (μmol/L).

IEM	Cases	Reference result	Reference value	Preliminary screening results [M (min∼max)]	Re-examination results [M (min∼max)]
PAHD	47	Phe	20–110	161.40 (112.00–1115.47)	158.03 (122.03–2194.20)
		Phe/Tyr	0.2–1.5	1.75 (0.78–13.98)	1.60 (0.67–43.16)
MIM	6	Met	8–50	55.97 (52.91–205.89)	83.45 (66.30–165.74)
		Met/Phe	0.15–0.9	1.35 (0.92–3.80)	1.71 (1.29–3.61)
MSUD	1	Leu	70–330	2942.15	6582.77
		Val	60–250	660.44	876.08
MMA	5	C3	0.5–5	14.39 (6.87–28.2)	9.75 (6.28–22.4)
		C3/C0	0–0.25	0.73 (0.26–0.86)	0.76 (0.19–2.13)
		C3/C2	0–0.22	0.52 (0.33–0.95)	1.34 (0.45–1.59)
3-MCCD	3	C4DC+C5OH	0.08–0.4	0.74 (0.73–7.50)	0.8 (0.78–12.73)
		(C4DC+C5OH)/C0	0–0.02	0.07 (0.03–0.56)	0.03 (0.03–4.90)
		(C4DC+C5OH)/C8	1–12	18.5 (6.08–375)	19.5 (11.43–1273)
PA	1	C3	0.5–5	7.96	14.45
		C3/C2	0–0.22	0.371	1.013
IVA	1	C5	0.04–0.3	4.56	7.37
		C5/C2	0–0.04	0.28	0.96
GA-I	1	C5DC+C6OH	0.04–0.25	2.54	2.37
		(C5DC+C6OH)/C8	0.5–5	63.5	237
2-MBAD	1	C5	0.04–0.3	0.45	1.23
		C5/C2	0–0.04	0.014	0.093
		C5/C3	0–0.45	0.086	1.255
SCADD	3	C4	0.09–0.55	0.92 (0.61–1.24)	0.96 (0.78–1.03)
		C4/C2	0–0.04	0.039 (0.037–0.059)	0.036 (0.028–0.093)
MCADD	2	C8	0.01–0.2	0.38 (0.35–0.41)	0.445 (0.44–0.45)
		C8/C2	0–0.02	0.03 (0.025–0.034)	0.029 (0.025–0.032)
PCD	2	C0	9–60	6.75 (5.47–8.03)	7.36 (6.04–8.68)

Note: Phe, phenylalanine; Tyr, tyrosine; Met, methionine; Leu, leucine; Val, valine; C0, free carnitine; C2, aceylcarnitine; C3, propionylcarnitine; C4, butyrylcarnitine; C5, isovalerylcarnitine; C8, octanoylcarnitine; C4DC+C5OH, succinylcarnitine/3-hydroxy-isovalerylcarnitine; C5DC+C6OH, glutarylcarnitine/3-hydroxy-hexanoylcarnitine.

### Genetic analysis in the confirmed cases

The gene detection results revealed 127 mutations of 11 IEMs-related genes in 69 confirmed cases. Four patients were diagnosed by specific biochemical markers. A total of 46 infants were tested by NGS sequencing, which detected 36 kinds of 89 mutation sites of the *PAH* gene: c.158G > A (28.09%) was the most common, followed by c.728G > A (10.11%) and c.688G > A (6.74%). Additionally, children carrying c.158G>A were diagnosed with mild hyperphenylalaninemia (120 μmol/L≤phe<360 μmol/L). According to the ACMG guideline, there were 70 pathogenic mutations, 13 probable pathogenic mutations, and 6 mutations of undetermined clinical significance. The *MAT1A* gene had seven different mutations sites: c.956C>A, c.227T>C, c.630C>T, c.361G>A, c.776C>T, c.765_768+4del, c.721_730delGTCTACCACC and allele frequencies was 14.29%. The *MMUT* and *MMACHC* genes were detected in 5 children with MMA. Among them, the *MMACHC* gene had four different pathogenic mutation sites. And the *MMUT* gene had five different pathogenic mutation sites and one probable pathogenic mutation site. A total of four kinds of five mutation sites were detected in the *ACADS* gene:c.625G > A (40%), c.1031A > G (20%), c.1054G > A (20%) and c.1130C > T (20%). The results of other disease-related genes is shown in Tables 4–6.

**TABLE 4:** Genetic test results of children diagnosed with Amino acid disorders.

Disease	Cases	Gene	Nucleotide variant	Amino acid variant	Pathogenic	Allele frequency (%)
Phenylalanine hydroxylase deficiency	46	*PAH*	c.158G>A	p.Arg53His	P	25 (28.09%)
			c.728G>A	p.Arg243Gln	P	9 (10.11%)
			c.688G>A	p.Val230Ile	P	6 (6.74%)
			c.1174T>A	p.Phe392Ile	LP	4 (4.49%)
			c.1238G>C	p.Arg413Pro	P	4 (4.49%)
			c.442-1G>A	—	P	4 (4.49%)
			c.331C>T	p.Arg111Ter	P	3 (3.37%)
			c.740G>T	p.GIy247Val	P	2 (2.25%)
			c.1262T>C	p.Ile421Thr	LP	2 (2.25%)
			c.320A>G	p.His107Arg	LP	2 (2.25%)
			c.355C>T	p.Pro119Ser	VUS	2 (2.25%)
			c.1316-1G>A	—	P	2 (2.25%)
			c.526C>T	p.Arg176Ter	P	1 (1.12%)
			c.977G>A	p.Trp326Ter	P	1 (1.12%)
			c.1256A>G	p.Gln419Arg	LP	1 (1.12%)
			c.1301C>A	p.Ala434Asp	P	1 (1.12%)
			c.694C>T	p.Gln232Ter	P	1 (1.12%)
			c.689T>C	p.Val230Ala	VUS	1 (1.12%)
			c.722G>A	p.Arg241His	P	1 (1.12%)
			c.1123C>G	p.Gln375Glu	LP	1 (1.12%)
			c.506G>A	p.Arg169His	LP	1 (1.12%)
			c.611A>G	p.Tyr204Cys	P	1 (1.12%)
			c.1194A>G	p.(=)	VUS	1 (1.12%)
			c.208_210del	p.Leu70del	P	1 (1.12%)
			c.782G>A	p.Arg261Gln	P	1 (1.12%)
			c.212G>A	p.Arg71His	LP	1 (1.12%)
			c.1199+1G>C	—	P	1 (1.12%)
			c.739G>C	p.Gly247Arg	P	1 (1.12%)
			c.311C>A	p.Ala104Asp	LP	1 (1.12%)
			c.907delT	p.Pro303Profs*12	P	1 (1.12%)
			c.722delG	p.Ala241Alafs*41	P	1 (1.12%)
			c.200C>G	p.Ser67Cys	VUS	1 (1.12%)
			c.842+2T>A	—	P	1 (1.12%)
			c.151G>C	p.Val51Leu	VUS	1 (1.12%)
			c.1197A>T	p.Val399Val	P	1 (1.12%)
			c.1162G>A	p.Val388Met	P	1 (1.12%)
Hypermethioninemia	5	*MAT1A*	c.956C>A	p.Ser319Tyr	VUS	1 (14.29%)
			c.227T>C	p.Met76Thr	VUS	1 (14.29%)
			c.630C>T	p.Asn210=	VUS	1 (14.29%)
			c.361G>A	p.Val121Ile	LP	1 (14.29%)
			c.721_730delGTCTACCACC	p.Val241fs	LP	1 (14.29%)
			c.765_768+4del	—	LP	1 (14.29%)
			c.776C>T	p.Ala259Val	P	1 (14.29%)

**TABLE 5:** Genetic test results of children diagnosed with organic acid disorders.

Disease	Cases	Gene	Nucleotide variant	Amino acid variant	Pathogenic	Allele frequency (%)
Methylmalonic acidemia	3	*MMUT*	c.914T>C	p.Leu305Ser	P	1 (16.67%)
			c.1106G>A	p.Arg369His	P	1 (16.67%)
			c.1663G>A	p.Ala555Thr	LP	1 (16.67%)
			c.632_636del	p.Glu211AlafsTer8	P	1 (16.67%)
			c.1677-1G>A	—	P	1 (16.67%)
			c.729_730insTT	p.Asp244LeufsTer39	P	1 (16.67%)
	2	*MMACHC*	c.445_446delTG	p.Cys149fs	P	1 (25%)
			c.567dupT	p.Ile190fs	P	1 (25%)
			c.609G>A	p.Trp203Ter	P	1 (25%)
			c.217C>T	p.Arg73Ter	P	1 (25%)
3-methylcrotonyl CoA carboxylase deficiency	3	*MCCC1*	c.1301T>C	p.Ile434Thr	VUS	1 (20%)
			c.1894C>T	p.Pro632Ser	VUS	1 (20%)
			c.1977G>A	p.Lys659Lys	LP	1 (20%)
			c.1679dupA	p.Asn560fs	P	1 (20%)
			c.841C>T	p.Arg281*	P	1 (20%)
Propionic acidemia	1	*PCCB*	c.1316A>G	p.Tyr439Cys	P	1 (50%)
			c.1364G>T	p.Trp455Leu	LP	1 (50%)
Isovaleric acidemia	1	*IVD*	c.1195G>C	p.Asp399His	LP	1 (100%)
2-methylbutyryl-CoA dehydrogenase deficiency	1	*ACADSB*	c.655G>A	p.Val219Met	LP	1 (100%)

**TABLE 6:** Genetic test results of children diagnosed with fatty acid oxidation disorders.

Disease	Cases	Gene	Nucleotide variant	Amino acid variant	Pathogenic	Allele frequency (%)
Short chain acyl-CoA dehydrogenase deficiency	3	*ACADS*	c.625G>A	p.Gly209Ser	VUS	2 (40%)
			c.1031A>G	p.Glu344Gly	LP	1 (20%)
			c.1054G>A	p.Ala352Thr	VUS	1 (20%)
			c.1130C>T	p.Pro377Leu	LP	1 (20%)
Medium chain acyl- CoA dehydrogenase deficiency	2	*ACADM*	c.74C>T	p.Thr25Ile	VUS	1 (25%)
			c.959C>A	p.Ser320*	P	1 (25%)
			c.928G>A	p.Gly310Arg	LP	1 (25%)
			c.991G>C	p.Glu331Gln	LP	1 (25%)
Primary carnitine deficiency	2	*SLC22A5*	c.254_264dupGGCTCGCCACC	p.I89Gfs*45	P	1 (33.33%)
			c.629A>G	p.Asn210Ser	LP	1 (33.33%)
			c.371A>G	p.Tyr124Cys	LP	1 (33.33%)

## Discussion

The incidences of genetic metabolic diseases varies dramatically in different countries and regions. In this study, the overall incidence of IEMs in Xinjiang was 1/1,476. Concerning domestic regional difference, it shared similarity in prevalence to Jining City (1/1,941) (Yang et al., 2020), which has been reported in the northern region, and showed higher trend than that in the southern regions, such as Hunan Province (1/5,282) (Yan et al., 2019), Nanjing City (1/2,835) (Sun et al., 2020), and Guiyang City (1/7,553) (Yang et al., 2022). Xinjiang is located in the northwest of China, where many ethnic groups live together, with unevenly distributed medical resources. There is a delayed genetic technology application in this region, restricting neonatal genetic and metabolic disease screening, diagnosis and treatment. Therefore, it is necessary to strengthen the IEMs screening work in this region.

PAHD is an amino acid metabolic disease with the highest prevalence (1/2,292) in Xinjiang, above the domestic average rate (1/10,000) (Ying and Luo, 2021). Moreover, the prevalence of HPA is higher in the northern part of China than in the southern part (Xiang et al., 2019). PAHD is a group of disorders in which phenylalanine (phe) metabolism is impaired by deficient enzyme phenylalanine hydroxylase or coenzyme tetrahydrobiopterin. Genetic testing was performed in 46 of the 47 cases of HPA diagnosed in this study, with 36 variants in *PAH* detected, of which c.158G > A (28.09%) was the hotspot mutation. It was consistent with the locus with the highest mutation frequency (24.7%) in Xuzhou (Zhou et al., 2021). Furthermore, children carrying this mutations site were diagnosed with mild hyperphenylalaninemia (120 μmol/L≤phe<360 μmol/L) and have been closely monitored for their blood phe levels. Patients with moderate phenylketonuria (360 μmol/L≤phe<1200 μmol/L) were managed with a low-Phe diet therapy. For classic phenylketonuria (phe≥1200 μmol/L), patients were given low Phe diet treatment and drug treatment, and their physical and intellectual development was no different from that of normal children of the same age. Six patients occurred hypermethioninemia due to methionine adenosyltransferase (MAT) defect, causing abnormal methionine metabolism. MAT is encoded by *MAT1A*, with four previously reported mutation sites, including c.769G > A, c.791G > A, c.874C > T and c.1070C > T (Feng et al., 2021). But this study observed seven unreported mutation sites, including c.956C > A, c.227T > C, c.630C > T, c.361G > A, c.776C > T, c.765_768+4del and c.721_730delGT CTACCACC, which enriched the gene database. One case of MSUD, despite early treatment and feeding with special formula milk, still presented symptoms such as feeding difficulties, convulsions, and a maple syrup smell in urine. The condition progressed rapidly and the child died in January.

MMA had the third highest incidence of all genetic metabolic diseases and ranked first among organic acid metabolic disease, with the prevalence rate of 1/21,548, remarkably higher than that in Italy (1/22,727) (Mussap et al., 2018), the United States (1/158,730) (Therrell Jr et al., 2014), and Japan (1/120,000) (Shibata et al., 2018) when compared with foreign countries. In this study, pure MMA and MMA combined with homocysteinemia accounted for 60% and 40%, respectively, which was similar to that reported in Guangzhou city (Xiong et al., 2019). Studies have shown that c.609G > A and c.567dupT are two common mutations of *MMACHC* gene in the Chinese population (Zhou et al., 2019). These mutation sites also appeared in the present study. However, the research results cannot define any genotype-phenotype correlation, and data need to be accumulated for further exploration. In addition, MCCD is a chromosomal recessive disorder caused by abnormalities in leucine metabolism, caused by *MCCC1* gene and *MCCC2* gene, respectively. Forsyth reported that the global incidence of MCCD was about 1/68,000–1/2,400 (Forsyth et al., 2016). So far, at least 66 *MCCC1* and 83 *MCCC2* mutations have been reported (Yang et al., 2015). The detection rate of MCCD was about 1/35,914 in Xinjiang population and all MCCD patients carried one or two *MCCC1* mutations. Although c.1301T > C and c.1894C > T are mutations of undetermined clinical significance, the children have obvious clinical symptoms. In a confirmed patient with PA, the *PCCB* gene c.1316A>G and c.1364G>T mutations were found. A patient with IVA, only one *IVD* gene c.1195G>C, which was causative mutation site, was detected from the mother. In a 2-MBAD case, only one site of a possible pathogenic mutation in the *ACADSB* gene was identified (c.655G>A). The confirmed patient with PA, GA-I, IVA and 2-MBAD were treated with low-protein diets, special formulas, and oral L-carnitine. During the six-month follow-up period, children showed normal growth and development.

In our study, SCADD is the most prevalent disease of fatty acid metabolic disorders (1/35,914), and the rate in Qingdao, China, was about 1/70,776 (Sun et al., 2019). Until now, more than 60 kinds of *ACADS* gene mutations have been reported, and most of them are missense mutations (Gregersen et al., 2008). Among them, the mutation sites c.511C>T and c.625G>A are more common in European and American countries and are hotspot mutations (Tonin et al., 2016). This is also reflected in this study. The incidence of MCADD was 1/100,000–1/30,000 in the United States (Grosse et al., 2006). In consistence with the above report, the incidence of MCADD in Xinjiang population is 1/53,871. Four variations of the *ACADM* gene were found by NGS: c.74C>T, c.959C>A, c.928G>A and c.991G>C. PCD is one of fatty acid metabolic disorders caused by mutations in the gene for the high-affinity carnitine transporter carnitine transporter protein (*SLC22A5*) on cell membranes. The prevalence of PCD is 1/40,000–1/120,000 in foreign countries and 1/8,938–1/45,000 in China (Lin et al., 2020). In this study, the prevalence of PCD in Xinjiang was 1/53,871. Two PCD patients showed an decrease of C0 detected by MS/MS. The genetic test detected three mutation sites on *SLC22A5* gene, including c.629A>G,c.371A>G and c.254_264dupGGCTCGCCACC.These children were given oral treatment with levocanidin, with good prognosis.

In summary, this study characterizes the disease spectrum of IEMs and genetic profiles on the basis of retrospective analysis of 107,741 newborns in Xinjiang in the past 6 years. This study has certain limitations, mainly manifested in the relatively high positive rate of the initial screening and the relatively low positive predictive value. Although tandem mass spectrometry technology has high sensitivity, it is also affected by factors such as the gestational age, weight and jaundice of newborns, which leads to a relatively high false positive rate. Artificial intelligence technology is highly efficient and accurate. By constructing a risk assessment model for IEMs in newborns, the risks of false positives and false negatives in the IEMs screening system can be reduced (Yang et al., 2021). Active IEMs screening, early treatment and long-term follow-up are pivotal for preventing and controlling birth defects and improving birth quality.

## Data Availability

The raw data supporting the conclusions of this article will be made available by the authors without undue reservation. As the data involves personal privacy, if necessary, you can contact the corresponding author for legal and legitimate reasons to obtain it.
